# Effect of the electric field at 50 Hz and variable intensities on biochemical markers in the honey bee’s hemolymph

**DOI:** 10.1371/journal.pone.0252858

**Published:** 2021-06-24

**Authors:** Paweł Migdał, Agnieszka Murawska, Paweł Bieńkowski, Aneta Strachecka, Adam Roman

**Affiliations:** 1 Department of Environment Hygiene and Animal Welfare, Wroclaw University of Environmental and Life Sciences, Wroclaw, Poland; 2 Telecommunications and Teleinformatics Department, Wroclaw University of Science and Technology, Wroclaw, Poland; 3 Institute of Biological Basis of Animal Production, Faculty of Biology, Animal Sciences and Bioeconomy, University of Life Sciences in Lublin, Lublin, Poland; King Khalid University, SAUDI ARABIA

## Abstract

The amount of artificial electromagnetic fields of various parameters in the honey bee’s environment increases globally. So far, it had been proven that exposure to an E-field at 50 Hz can cause changes in bee’s behavior, alter the activity of proteases, and enzymatic antioxidants. Due to the potentially harmful effect of this factor on honey bees, we decided to investigate the activity of aspartate aminotransferase (AST), alanine aminotransferase (ALT), and alkaline phosphatase (ALP), and the concentration of albumin and creatinine in bee’s hemolymph after exposure to 50 Hz E-field. Honey bee workers were placed in wooden cages (200 × 150 × 70 mm) and exposed to the 50 Hz E-field with the intensity of <1, 5.0, 11.5, 23.0, or 34.5 kV/m for 1, 3, 6, or 12h. A homogeneous 50 Hz E-field was generated in the form of a plate capacitor. Hemolymph samples for analysis were taken immediately after the end of exposure to the E-field from 100 bees from each group. According to our study, the activity of AST, ALT, and ALP in honey bees’ hemolymph decreased after exposure to 50 Hz E-field with various intensities. The decrease in AST, ALT, and ALP activity intensified with prolonged exposure time. 50 Hz E-field may cause the impairment of crucial metabolic cycles in the honey bees’ organism (such as the citric acid cycle, ATP synthesis, oxidative phosphorylation, β-oxidation). Moreover, exposure to E-Field altered the concentration of creatinine and albumin, which are important non-enzymatic antioxidants. Such changes may indicate a disturbance in protein metabolism and increased muscle activity.

## Introduction

The dynamically developing civilization causes an increased demand for electricity [1, 2]. Consequently, the amount of artificial electromagnetic fields of various parameters in the honey bee’s environment increases. The presence of bees, the most efficient pollinators, has a positive effect on maintaining biodiversity and increasing agricultural yields [3]. It is important to investigate each of the factors that potentially threaten this insect.

In recent years, there has been a growing interest in urban beekeeping [4]. Locating the bees in urban environments makes it hard for them to avoid exposure to E-field at different frequencies and intensities. Apiaries are placed near high-voltage lines and in cities [5], where avoiding electromagnetic fields would be impossible or very difficult. So far it has been proven that a 50 Hz electric field (E-field) with the intensity of 5.0, 11.5, 23.0, 34.5 kV/m and different exposure time changes bee activity in laboratory conditions. Bees after exposition to E-field with these parameters displayed self-grooming and contact between other individuals less frequently compared to the control bees [6, 7]. After acute exposure to a 50Hz low-frequency electromagnetic field, bees’ ability to learning, flying towards feed, and feeding or walking was reduced [8–10]. 60 Hz electromagnetic field with the intensity above 150 kV/m caused vibrations of the bee body [11]. Furthermore, exposure to 50 Hz E-field with the intensity of 5.0 kV/m, 11.5 kV/m, 23.0 kV/m, 34.5 kV/m for 1, 3, 6, or 12h changed the activity of enzymatic antioxidants (superoxide dismutase (SOD), catalases (CAT)), and acidic, neutral, and alkaline proteases [7, 12, 13].

However, knowledge of the impact of the electromagnetic field on honey bee physiology is limited. All biochemical barriers and mechanisms, such as, among others, antioxidant and proteolytic systems, are designed to maintain homeostasis or protect the organism from pathogens. An important element of this system are enzymes responsible for the detoxification of the organism (AST, ALT, ALP, GGTP, and bilirubin). Changes in the activity of these indicators indicate increased inflammatory or defense processes [14]. This system is complemented by non-enzymatic antioxidants which donate their electrons to free radicals. As a result, the possibility of oxidation of other components is blocked. They protect cells against free radical reactions. They can be endogenous (melatonin, glutathione, estrogen, albumin) or exogenous (vitamins, coenzyme Q10, some elements) [15, 16]. The indicator characterizing the increased activity of the organism is also the level of creatinine, which is a product of the breakdown of creatine phosphate from the metabolism of muscles and proteins. It is released by the body at a constant rate. Disturbance of its level means increased muscle work or increased protein metabolism [17]. Since the body’s defense system is very complex, any disturbance in its working can make the insect more susceptible to diseases and environmental stressors [13]. Due to the potentially harmful effect of E-field on bees, we decided to investigate the activity of enzymatic biochemical markers: aspartate aminotransferase (AST), alanine aminotransferase (ALT), and alkaline phosphatase (ALP), and the concentration of non-enzymatic antioxidant: albumin and creatinine in bee’s hemolymph after exposure to 50 Hz E-field with the intensity of 5.0 kV/m, 11.5 kV/m, 23.0 kV/m, 34.5 kV/m for 1, 3, 6 or 12 h.

## Material and methods

### Rearing worker bees

Four weeks before the experiment 10 mated queens were introduced into 10 queenless honey bee (*Apis mellifera carnica* L.) colonies. After mothers start laying eggs each queen was kept in one isolator with empty Dadant comb (435 × 300 mm). The combs with eggs were marked and left in a bee colony. On the 20th day of bee development, the combs were transferred to an incubator with conditions like in bee hive (temperature of 34.4°C ± 0.5°C and relative humidity of 70% ± 5%) to emerge. Honey and bee pollen were provided *ad libitum* until the bees were transferred to the cages.

### Experimental design

1-day-old honey bee workers were placed in wooden cages (200 × 150 × 70 mm) with two feeders (5 ml each). Each cage contained 100 workers. Each of the experimental and control groups consisted of 10 cages. Sucrose solution at a concentration of 1 mol/dm^3^ was provided to bees *ad libitum*. In the experimental groups 2-day-old worker bees were exposed to the 50 Hz E-field with the intensity of 5.0, 11.5, 23.0, or 34.5 kV/m for 1, 3, 6, or 12h. The measured value of E-field in the area where the control groups were kept was <1.0 kV/m and the control groups were marked with the letter C and number of hours of exposure corresponding to the experimental groups’ exposure duration.

The selected E-field parameters result from the possible exposure of the honey bee to the electromagnetic field in nature. 50 Hz is a widely used power frequency in the world. Worker bees were flying at a height of about 2 meters near the power line is exposed to an E-field with an intensity up to 2–10 kV/m. High obstacles in the worker’s way caused flight at a high of about five or more meters, then the bee is exposed to an E-field intensity even up to 12–15 kV/m. The time which bees can spend in the environment while searching the feed varies between 1 to up to 6h. 12h exposure was chosen to check linearity of the phenomenon.

### E-field setup

**Fig 1:**
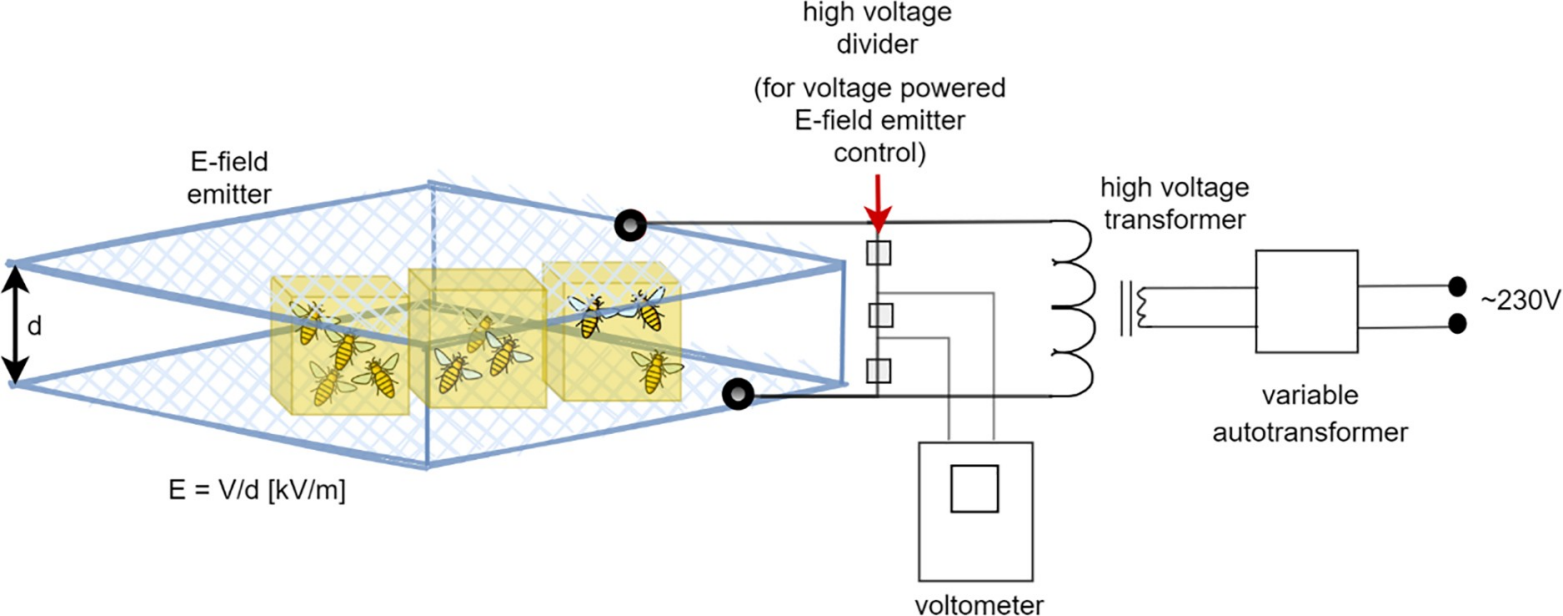
Exposure of bees to the E-field at 50 Hz and variable intensities in laboratory conditions.

A homogeneous 50 Hz E-field was generated in the exposure system in the form of a plate capacitor as per Migdał et al. [13]. The field intensity was fixed to 5.0 kV/m, 11.5 kV/m, 23.0 kV/m, or 34.5 kV/m. Changes in homogeneity and stability of E-field intensity were no higher than ± 5% in the emitter in which the bees were exposed during the experiment. Field intensity and homogeneity in the test area were verified by the measurements made by an LWiMP accredited testing laboratory using the ESM-100-meter No. 972153 with the calibration certificate LWiMP/W/070/2017 dated 15/02/2017 issued by the accredited calibration laboratory PCA AP-078. The measurements were done in the points of a 10 x 10 x 5cm, 3 mesh inside an empty emitter. The stability of the generated E-field was maintained by permanently monitoring the voltage applied to the exposure system using a control circuit (Fig 1).

### Hemolymph analyses

Hemolymph samples were taken immediately after the end of exposure to the E-field from 100 bees from each group. Material was collected from the appropriate control group at the same time to keep the same time intervals. To collect hemolymph, we removed the bee’s antennae with sterile tweezers and gently pressed the bee’s body as per Migdał et al. [18]. Hemolymph samples were stored in a 20 μl glass capillary without anticoagulant at -80°C. The activities of enzymatic biochemical markers: aspartate aminotransferase (AST), alanine aminotransferase (ALT), and alkaline phosphatase (ALP) in bee’s hemolymph were measured with the kinetic method using monotests from Cormay (Lublin, Poland) according to the manufacturer’s procedure.

#### Alanine aminotransferase (ALT)

ALT reagent composition at pH 7.8 was prepared from the following compounds: 2-ketoglutarate (13 mmol/L), L-alanine (440 mmol/L), NADH (0.10 mmol/L), LDH (1800 U/L), Tris buffer (97 mmol/L) and EDTA (5.0 mmol/L). Then, ALT reagent composition was heated to 37°C. 100 μl of ALT reagent composition was poured into 10 μl of sample / hemolymph, mixed on vortex for 3–5 seconds, heated for 30 s. at 37°C and the absorbance at 340 nm was measured during T 0, then 1, 2 and 3 minutes after incubation.

#### Aspartate aminotransferase (AST)

ALT reagent composition at pH 8.1 was prepared from the following compounds: 2- ketoglutarate (13 mmol/L), L-aspartate (220 mmol/L), LDH (1200 U/L), MDH (90 U/L), NADH (10 mmol/L), Tris buffer (88 mmol/L) and EDTA (5.0 mmol/L). Then, AST reagent composition was heated to 37°C. 100 μl of AST reagent composition was poured into 10 μl of sample / hemolymph, mixed on vortex for 3–5 seconds, heated for 30 seconds. at 37°C and the absorbance at 340 nm was measured during T 0, then 1, 2 and 3 minutes after incubation.

#### Alkaline phosphatase (ALP)

ALP reagent composition was prepared from the following compounds: 2-amino-2-methyl-1- propanol (900mmol/L), magnesium acetate (1.6 mmol/L), zinc sulphate (0.4 mmol/L) and HEDTA (2.0 mmol/L). Then, ALP reagent composition was heated to 37°C. 100 μl of ALP reagent composition was poured into 2 μl of sample / hemolymph, mixed on vortex for 3–5 seconds, heated for 60 s. at 37°C. Next, 20 μl 4-NPP (16.0 mmol/L) was added to the sample/solution, mixed on vortex for 5 second and heated for 60 s. at 37°C. The absorbance was measured at 405 nm in time T 0 and then 1, 2 and 3 minutes after incubation.

Activities of ALT, AST and ALP were calculated according to the formula:

*Activity*_*ALT/AST/ALP*_ *= ΔAbs/min x F*
*F*_*ALT/AST*_ *= (TV x 1000)/(6*.*3 x SV x P)*
*F*_*ALP*_ *= (TV x 1000)/(18*.*8 x SV x P)*
*ΔAbs/min = ((A2-A1) + (A3-A2) + (A4-A3))/3*

where:

*A1*, *A2*, *A3*, *A4*—individual readings of the absorbance values for the samples
*TV*—total volume of the reaction mixture
*SV*—sample volume used for the reaction
*P*—optical path length of the cuvette
*6*.*3*—absorbance factor for dihydronicotinamide adenine dinucleotide (NADH; at 340-nm
wavelength)
*18*.*8*—absorbance factor for 2,4-dinitrophenol (2,4-DNP)

Non-enzymatic antioxidant (albumin, creatinine) concentrations in bee’s hemolymph were determined with the colorimetric method using monotests from Cormay (Lublin, Poland) according to the manufacturer’s procedure.

#### Albumin (Alb) concentration

To 2 μl sample/hemolymph was added 100 μl of Reaction Mix (0.4 μl Dilute Bromocresol Green–BCG + 3.6 μl ddH 2 0 + 96 μl Albumin Assay Buffer). To control was added 100 μl of Background Reaction Mix. All samples were incubated at 25°C for 20 minutes protected from light. Absorbance was measured at 620 nm.

Albumin concentration was calculated as:

*Alb = B/V x D*

Where:

*B*–amount of Alb in the sample well calculated from standard curve
*V*–sample volume added in the well (μl)
*D*–sample dilution factor if sample is diluted to fit within the standard curve range (prior to
reaction well set up).

#### Creatinine concentration

To 15 μl sample/hemolymph was added 150 μl Alkaline Picrate Solution (6 ml of Creatinine Surfactant + 10 ml of Creatinine Color Reagent + 3.6 ml of Creatinine NaOH) and incubated for 10 minutes at room temperature. The absorbance was measured at 492 nm (Initial absorbance reading–I abs). Then, 5 μl of acid solution was added and incubated on a shaker for 20 minutes at room temperature. The absorbance was measured at 492 nm (Final absorbance reading–F abs).

Creatinine concentration was calculated as:

*Abs*. *= F abs—I abs*
*Creatinine = (Abs*.*– 0*.*0001)/0*.*041*

### Data evaluation

The statistical significance of data within and between groups was first determined by the non-paramteric Kruskal Wallis test. To see which groups differ from each other, we chose Dunn’s with Bonferroni correction post hoc rank sum comparision using the package “pgirmess” for “krus-calmc” function. For all tests, RStudio and a significance level of α = 0.05 were used.

## Results

### Activity of enzymatic biochemical markers (AST, ALT, ALP) in bee’s hemolymph

AST, ALT, ALP activity in the hemolymph of the bees in the control groups did not differ (Table 1). Activities of these biochemical markers in each of the experimental groups were lower than in the control groups. The activity of AST, ALT, and ALP in the experimental groups was the lower the longer the exposition time. Differences between AST activity in the control groups and all experimental groups were statistically significant (Table 1). The lowest activity of all three biochemical markers was noticed in the bees treated with the highest E-field intensity (i.e. 34.5 kV/m) for 12h. The highest activity of AST, ALT, ALP among the experimental groups was noticed in the 11.5 kV/m 1h group.

**Table 1:** Comparison of the selected biochemical markers in the honey bee’s hemolymph as a function of exposure time and E-field intensity.

E-field intensity (kV/m)	Exposure time (h)	Selected biochemical markers (±SD)				
		AST (U/liter)	ALT (U/liter)	ALP (U/liter)	Albumin (g/dl)	Creatinine (μmol/liter)
< 1.0 control groups	1	80.52 (±0.26)^a^	52.42 (±0.31)^a^	25.41 (±0.28)^a^	0.054 (±0.003)^a^	55.53 (±0.27)^a^
	3	80.72 (±0.40)^a^	52.25 (±0.47)^a^	25.37 (±0.41)^a^	0.054 (±0.003)^a^	55.63 (±0.16)^a^
	6	80.58 (±0.36)^a^	52.45 (±0.25)^a^	25.44 (±0.26)^a^	0.054 (±0.003)^a^	55.46 (±0.27)^a^
	12	80.54 (±0.27)^a^	52.44 (±0.29)^a^	25.49 (±0.32)^a^	0.054 (±0.004)^a^	55.49 (±0.24)^a^
5.0	1	70.42 (±0.27)^b^	50.40 (±0.40)^a^	22.52 (±0.22)^b^	0.084 (±0.003)^b^	52.62 (±0.22)^b^
	3	68.52 (±0.25)^b^	49.41 (±0.44)^a^	21.15 (±0.54)^b^	0.094 (±0.004)^b^	51.97 (±0.54)^b^
	6	67.53 (±0.30)^bc^	47.59 (±0.34)^b^	19.75 (±0.50)^b^	0.120 (±0.026)^b^	51.00 (±0.47)^b^
	12	66.41 (±0.48)^c^	46.54 (±0.38)^b^	17.65 (±0.38)^c^	0.190 (±0.006)^c^	50.23 (±0.21)^bc^
11.5	1	72.55 (±0.57)^b^	50.47 (±0.35)^a^	23.43 (±0.43)^ab^	0.060 (±0.050)^a^	55.52 (±0.26)^a^
	3	53.33 (±0.40)^d^	48.34 (±0.32)^ab^	14.40 (±0.50)^d^	0.074 (±0.004)^ab^	50.51 (±0.34)^b^
	6	52.40 (±0.30)^d^	46.31 (±0.41)^b^	12.68 (±0.16)^d^	0.084 (±0.002)^b^	48.50 (±0.26)^c^
	12	48.43 (±0.32)^d^	39.34 (±0.54)^c^	10.51 (±0.30)^e^	0.094 (±0.002)^b^	42.46 (±0.30)^d^
23.0	1	70.59 (±0.32)^b^	47.38 (±0.47)^b^	20.74 (±0.10)^b^	0.055 (±0.003)^a^	55.58 (±0.24)^a^
	3	40.66 (±0.42)^e^	45.54 (±0.55)^b^	13.46 (±0.25)^d^	0.131 (±0.014)^b^	50.41 (±0.17)^bc^
	6	38.45 (±0.29)^e^	42.37 (±0.48)^c^	9.54 (±0.41)^e^	0.135 (±0.004)^b^	48.49 (±0.24)^c^
	12	32.51 (±0.38)^f^	32.59 (±0.48)^d^	8.41 (±0.30)^e^	0.160 (±0.004)^c^	42.45 (±0.31)^d^
34.5	1	65.23 (±0.21)^c^	45.41 (±0.44)^b^	19.48 (±0.32)^b^	0.060 (±0.003)^a^	55.50 (±0.26)^a^
	3	36.53 (±0.34)^f^	42.53 (±0.30)^c^	11.49 (±0.30)^d^	0.193 (±0.015)^c^	50.51 (±0.26)^bc^
	6	30.44 (±0.25)^f^	41.36 (±0.53)^c^	10.26 (±0.90)^d^	0.240 (±0.024)^d^	48.57 (±0.18)^c^
	12	18.38 (±0.37)^g^	30.50 (±0.27)^d^	5.44 (±0.30)^f^	0.233 (±0.015)^d^	42.50 (±0.28)^d^
p-value		2.2x10^-16^	2.2x10^-16^	2.2x10^-16^	2.2x10^-16^	2.2x10^-16^
Chi-squared		1884.9	1869	1879.7	1804.8	1801.6

The average levels of selected biochemical markers in the honey bee’s hemolymph after bees exposure to the E-field at 50 Hz and intensity of 5.0 kV/m, 11.5 kV/m, 23.0 kV/m, or 34.5 kV/m for 1, 3, 6, or 12h. Standard deviation is shown in round brackets. Different letters (a,b,c,…) indicate statistical differences at the p ≤ 0.05 significance level between various exposure times and E-field intensity.

### Non-enzymatic antioxidant concentration (creatinine and albumin) in bee’s hemolymph

The concentrations of albumin and creatinine within all control groups were not different (Table 1). The albumin concentration in the hemolymph of bees treated with the E-field intensity of 5.0 and 11.5 kV/m was the higher the longer the exposition time. The concentration of creatinine in the experimental groups was the lower the longer the exposition time. One hour of exposure to the E-field with the intensity of 11.5, 23.0, and 34.5 did not cause statistically significant changes in the concentration of albumin and creatinine (Table 1). After 3 and more hours of exposure to the E-field, the concentration of creatinine in the experimental groups was lower than in the control groups, while the concentration of albumin was higher compared with the control groups.

## Discussion

Studies of the effects of E-fields at different frequencies and intensities on various groups of insects indicate their significant role in altering insect behavior and physiology. Recent studies indicate behavioral changes in migratory locusts (*Locusta migratoria* L.), which were exposed to electromagnetic fields at extremely low frequencies. Authors observed that the number of wing beats increased with electromagnetic field intensity [19]. Behavioral changes were also observed in honey bee exposed to E-field at 50Hz and different frequencies and exposure time. Insects under influence of E-field were less active than control group [6]. In addition, behavioral changes were compared with the activity of the proteases in the hemolymph of the bees [20]. Results of Shepherd et al. [8, 10] indicate that exposure to extremely low frequency (ELF) electromagnetic fields (EMFs) reduced aversive learning, alter flight dynamics, reduce the success of foraging flights towards food sources, and feeding, and increased level of aggression. Authors assumed that ELF electromagnetic fields at 50 Hz emitted from power lines may be an environmental stressor for honey bees, possibly altering their cognitive and motor skills, which could reduce their ability to pollinate crops [8]. According to our study, the activity of aspartate aminotransferase (AST), alanine aminotransferase (ALT), and alkaline phosphatase (ALP) in honey bees’ hemolymph after exposure to 50 Hz E-field with the intensity of 5.0, 11.5, 23.0, or 34.5 kV/m for 1, 3, 6, or 12h were significantly lower in comparison to the control bees. In the hemolymph of healthy honey bees, the activity of ALT, AST, and ALP increases during the aging process [21]. Moreover, the decrease in the activity of ALT, AST, and ALP in honey bee organisms is considered a negative phenomenon and is caused by various types of harmful factors [21–24].

The AST and ALT are transaminases known to be crucial links between carbohydrate and protein metabolism. The activity of these enzymes alters during different physiological or pathological conditions [25]. The AST takes part in the conversion of aspartate and α-ketoglutarate to oxaloacetate (and vice versa) during the citric acid cycle and transamination [26]. The ALT catalyzes the transfer of the amino group from L-alanine to α-ketoglutarate. The products of this reaction are pyruvate and L-glutamate [27]. The ALP through dephosphorylation hydrolyzes phosphate groups in various molecules such as nucleotides, proteins, and alkaloids in alkaline or acidic conditions [26]. This enzyme can be an indicator of digestion efficiency and transportation of nutrients between midgut, hemolymph, and fat bodies [26, 28, 29]. Reduce activity of AST, ALT, and ALP in honey bee’s hemolymph may indicate that these enzymes were not released and/or were blocked by harmful factors [21]. Results of our study show that a 50 Hz E-field with the intensity of 5.0, 11.5, 23.0, or 34.5 kV/m for 1, 3, 6, or 12h by decreasing AST, ALT, and ALP activity may cause the disturbance in crucial metabolic cycles (such as the citric acid cycle, ATP synthesis, oxidative phosphorylation, β-oxidation).

In our study, after exposure to the 50 Hz E-field with the intensity of 5.0, 11.5, 23.0, or 34.5 kV/m for 1, 3, 6, or 12h the concentration of creatinine in the hemolymph of bees from experimental groups was lower than in the control groups, while the concentration of albumin was higher compared to the control groups.

In studies of Koziorowska et al. [30] the honeybees were treated with EMF at 50 Hz and magnetic induction of 1.6 mT for 2, 6, 12, 24 and 48 hours. EMF exposure for more than 2 hours caused changes in the structure of chemical compounds, especially in the IR region corresponding to DNA, RNA, phospholipids and protein vibrations. The effect of EMF depended on the duration of exposure. In our previous study [12, 13], we investigated the influence of the E-field at the intensity of 5.0, 11.5, 23.0, or 34.5 kV/m on ferric ion reducing antioxidant power (FRAP) and the activity of antioxidant enzymes: superoxide dismutase (SOD) and catalase (CAT). The level of FRAP did not significantly differ between groups (except for exposure to 50 Hz E-field with the intensity of 11.5 and 34.5 kV/m for 12h). The activity of SOD and CAT increased or decreased in the experimental groups. These changes may indicate dysregulation of the antioxidant system, however, no relationship with the change in E-field intensity or duration of exposure was observed. Our results indicate that E-Field may affect the honey bee antioxidant system by altering the concentration of non-enzymatic antioxidants in the hemolymph. Such changes may indicate a disturbance in protein metabolism and increased muscle activity.

## Conclusions

In our study, the activity of AST, ALT, and ALP in honey bees’ hemolymph decreased after exposure to 50 Hz E-field with various intensity. Reduce activity of these enzymes in honey bee’s hemolymph may indicate that these enzymes were not released and/or were blocked by a harmful factor. 50 Hz E-field with the various intensities may cause the impairment of crucial metabolic cycles in the honey bees’ organism (such as the citric acid cycle, ATP synthesis, oxidative phosphorylation, β-oxidation). Moreover, exposure to 50 Hz E-Field with various intensities may affect honey bees by altering the concentration of creatinine and albumin, which are important non-enzymatic antioxidants. The changes in AST, ALT and ALP activity intensified with prolonged exposure time regardless of the E-field intensity. An interesting issue for further research is whether changes in investigated biochemical parameters after exposure to E-field persist, and for how long.
